# The Dark Side of the Moon: Meta-analytical Impact of Recruitment Strategies on Risk Enrichment in the Clinical High Risk State for Psychosis

**DOI:** 10.1093/schbul/sbv162

**Published:** 2015-11-20

**Authors:** Paolo Fusar-Poli, Frauke Schultze-Lutter, Marco Cappucciati, Grazia Rutigliano, Ilaria Bonoldi, Daniel Stahl, Stephan Borgwardt, Anita Riecher-Rössler, Jean Addington, Diana O. Perkins, Scott W. Woods, Thomas McGlashan, Jimmy Lee, Joachim Klosterkötter, Alison R. Yung, Philip McGuire

**Affiliations:** ^1^Department of Psychosis Studies, King’s College London, Institute of Psychiatry, Psychology and Neuroscience, London, UK;; ^2^OASIS Service, South London and the Maudsley NHS Foundation Trust, London, UK;; ^3^Department of Brain and Behavioural Sciences, University of Pavia, Pavia, Italy;; ^4^University Hospital of Child and Adolescent Psychiatry and Psychotherapy, Department of Child and Adolescent Psychiatry, University of Bern, Bern, Switzerland;; ^5^Department of Biostatistics, Institute of Psychiatry Psychology and Neuroscience, King’s College London, London UK;; ^6^Department of Psychiatry (UPK), University of Basel Psychiatric Clinics, Basel, Switzerland;; ^7^Department of Psychiatry, University of Calgary, Calgary, Alberta, Canada;; ^8^Department of Psychiatry, University of North Carolina, Chapel Hill, NC;; ^9^Department of Psychiatry, Yale University, New Haven, CT;; ^10^Department of General Psychiatry, Institute of Mental Health, Singapore, Singapore;; ^11^Department of Psychiatry and Psychotherapy, University of Cologne, Cologne, Germany;; ^12^Institute of Brain, Behaviour and Mental Health, University of Manchester, Manchester, UK.

**Keywords:** psychosis, prevention, CAARMS, SIPS, schiz-ophrenia, meta-analysis/risk

## Abstract

**Background:**

The individual risk of developing psychosis after being tested for clinical high-risk (CHR) criteria (posttest risk of psychosis) depends on the underlying risk of the disease of the population from which the person is selected (pretest risk of psychosis), and thus on recruitment strategies. Yet, the impact of recruitment strategies on pretest risk of psychosis is unknown.

**Methods:**

Meta-analysis of the pretest risk of psychosis in help-seeking patients selected to undergo CHR assessment: total transitions to psychosis over the pool of patients assessed for potential risk and deemed at risk (CHR+) or not at risk (CHR−). Recruitment strategies (number of outreach activities per study, main target of outreach campaign, and proportion of self-referrals) were the moderators examined in meta-regressions.

**Results:**

11 independent studies met the inclusion criteria, for a total of 2519 (CHR+: *n* = 1359; CHR−: *n* = 1160) help-seeking patients undergoing CHR assessment (mean follow-up: 38 months). The overall meta-analytical pretest risk for psychosis in help-seeking patients was 15%, with high heterogeneity (95% CI: 9%–24%, *I* ^2^ = 96, *P* < .001). Recruitment strategies were heterogeneous and opportunistic. Heterogeneity was largely explained by intensive (*n* = 11, β = −.166, *Q* = 9.441, *P* = .002) outreach campaigns primarily targeting the general public (*n* = 11, β = −1.15, *Q* = 21.35, *P* < .001) along with higher proportions of self-referrals (*n* = 10, β = −.029, *Q* = 4.262, *P* = .039), which diluted pretest risk for psychosis in patients undergoing CHR assessment.

**Conclusions:**

There is meta-analytical evidence for overall risk enrichment (pretest risk for psychosis at 38monhts = 15%) in help-seeking samples selected for CHR assessment as compared to the general population (pretest risk of psychosis at 38monhts=0.1%). Intensive outreach campaigns predominantly targeting the general population and a higher proportion of self-referrals diluted the pretest risk for psychosis.

**“**It is an epidemiological fact that the degree of risk associated with meeting any screening criteria depends on the prevalence of the condition in the population being studied” [Yung et al 2008]^1 (p16)^

## Introduction

**Fig. 1.**
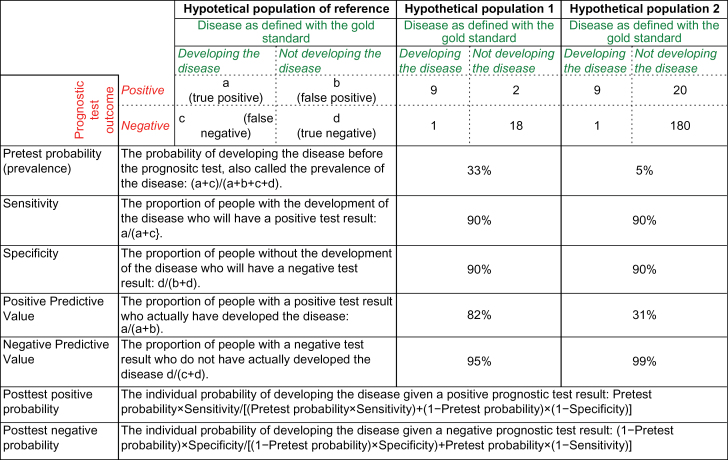
Pretest and posttest probability in prognostic tests.

Pretest probability and posttest risk of psychosis index an individual’s probabilities to develop a psychotic disorder before and after the clinical high risk (CHR thereafter, see supplementary material 1 for details) assessment, respectively (http://www.cebm.net/pretest-probability/). To be clinically useful, the results of the CHR assessment should substantially increase the difference between pre- and post-assessment risk of psychosis. Posttest risk, in turn, can be positive or negative, depending on whether the CHR assessment falls out as a positive test (at risk, CHR+) or a negative test (not at risk, CHR−), respectively. Positive predictive values and negative predictive values may be used to estimate the posttest probabilities.^2^ However, their values could assume a different relevance depending on the population investigated,^2^ and sampling biases across different populations may impact their clinical usefulness. Figure 1 illustrates these concepts. A hypothetical prognostic test with 90% specificity and 90% sensitivity, applied in a hypothetical population 1 characterized by a disease prevalence of 33% will deliver a positive predictive value of 82% and a negative predictive value of 95%. The same test, with the same 90% specificity and sensitivity, when applied in another hypothetical population 2 with a 5% prevalence of the disease, will yield a positive predictive value of 31% and a negative predictive value of 99%. Indeed, on the basis of the Bayes theorem,^3^ the posttest probability in a particular clinical situation depends not only on the test’s characteristics (sensitivity and specificity) but also on the patient’s probability to develop the disease before the test result is known (pretest probability).^3^ What this means in CHR practice is that the usefulness of CHR assessment (posttest risk of psychosis) for an individual patient depends on the underlying risk of psychosis in the population being tested (pretest risk of psychosis).^2^

**Fig. 2.**
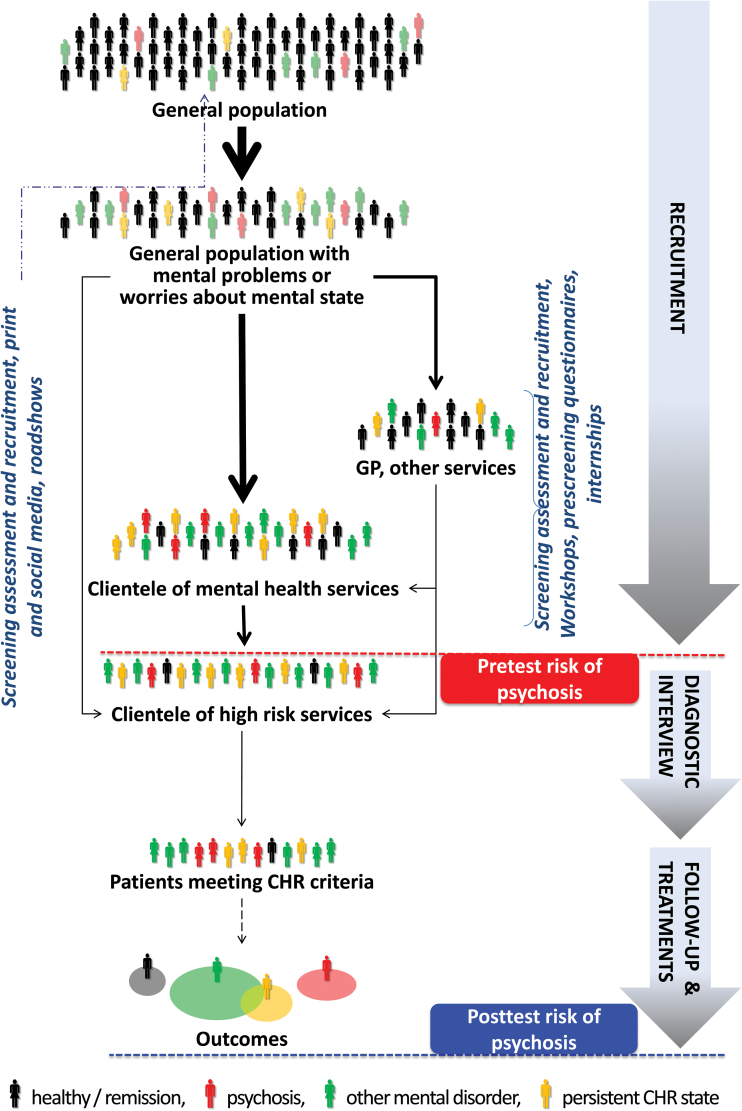
Illustration of hypothetical recruitment processes and, relatedly, risk enrichment of pretest risk of psychosis in clinical high-risk (CHR) samples, adapted from.^44^ In the general population, mental problems, mainly anxiousness, depressiveness^45^ and family/partner problems,^38^ and/or worries about one’s mental state may lead to help-seeking. Thereby, outreach campaigns targeting the general population might lead to seeking help directly at high-risk services (self-referrals) but might also unspecifically increase symptom awareness and, relatedly, worries about one’s mental state and help-seeking, thus potentially resulting in a risk dilution. Outreach campaigns targeting (mental) health providers may lead to selective referrals from these sources suspected to possibly suffer from a CHR state; thereby pretest risk enrichment will likely be highest in referrals from mental health professionals.^17^

These theoretical arguments are of critical relevance for the prognostic assessment of CHR patients. International instruments have been validated, and their interrater reliability has been demonstrated,^4–6^ along with their overall psychometric properties (ie, sensitivity and specificity^7^). A few meta-analyses have summarized the positive predictive values of the CHR prognostic testing against gold standard (psychosis onset as established by international ICD/DSM manuals).^8–10^ However, such predictive values should be used as estimates of posttest risk of psychosis only in a very specific population of help-seeking subjects recruited in high-risk services with a determinate pretest risk of psychosis. Unfortunately, such pretest risk of psychosis is currently unknown, limiting the generalizability and reproducibility of CHR findings to other populations. Therefore, the recent EPA guidelines recommend restricting the CHR assessment to individuals already distressed by mental problems and seeking help for them or individuals seeking clarification of their current risk for psychosis.^9^ However, despite these recommendations, recruitment strategies adopted by high-risk services are heterogeneous and not standardized (figure 2). Thus, the impact of sampling biases associated with such heterogeneous recruitment strategies on pretest risk of psychosis is completely unknown.

For example, recent studies^11^ and meta-analyses^8,9^ reported considerably lower transition risks as compared to earlier CHR studies. It was suggested that this risk dilution was caused by changes in referral pathways^11^ and inclusion of younger age groups^9,12^ and, consequently, in changes of the populations (ie, pretest risk of psychosis) from which CHR patients are selected. Thus, recruitment strategies might play a crucial role in the accuracy of predicting psychosis onset using CHR criteria. The first study suggesting that the process of recruiting CHR patients “is a major challenge for prevention of psychotic disorders”^13^ was published approximately 15 years ago. Only few descriptive reports^14–19^ and even less original studies^1,11,20^ have addressed their potential impact on the pretest risk of psychosis.

Recruitment strategies are of clinical and economic significance, as the psychopathological examination for determining if an individual meets CHR criteria requires several hours of time of extensively trained mental health professionals per case.

We present here the first meta-analysis investigating the magnitude and consistency of pretest risk of psychosis in patients assessed at high-risk services. We hypothesized that pretest risk of psychosis would be significantly higher in these help-seeking samples than in the general population.^21^ We additionally examined with meta-regressions the impact of recruitment strategies on the pretest risk of psychosis in help-seeking patients selected to undergo CHR interview. We hypothesized that type of recruitment strategy would impact the pretest risk of psychosis of patients selected for CHR assessment.

## Methods

### Search Strategy

Two investigators (M.C., G.R.) conducted 2-step literature searches. First, the Web of Knowledge database was searched, incorporating both the Web of Science and MEDLINE®. The search was extended until March 2015, including abstracts in English language only. The electronic research adopted several combinations of the following keywords: “at risk mental state,” “psychosis risk,” “prodrome,” “prodromal psychosis,” “ultra-high risk,” “high risk,” “help-seeking,” “referral,” “recruitment,” “psychosis prediction,” “psychosis onset,” and names of possible CHR instruments. Second, we used Scopus® to investigate citations of possible previous reviews/meta-analyses on recruitment strategies and transition risks, respectively, and a manual search of the reference lists of retrieved articles. Articles identified through these 2 steps were then screened for the selection criteria on basis of abstract reading. The articles surviving this selection were assessed for eligibility on the basis of full-text reading, following the MOOSE checklist (supplementary table 1).^22^

### Selection Criteria

Studies were eligible for inclusion if the following criteria were fulfilled: (*a*) were original articles, written in English or German; (*b*) examined with an established CHR instrument the same pool of help-seeking patients selected for CHR assessment^5,23–27^; (*c*) reported on risk of transition to psychosis of both CHR+ and CHR− patients; (*d*) reported on type of recruitment strategies. When relevant data were not directly presented, they were indirectly extracted from associated data. Additionally, we contacted all corresponding authors to request additional data when needed, in particular with respect to the type of recruitment strategies and moderators.

Exclusion criteria were: (*a*) abstracts, pilot datasets, and papers in languages other than those above; (*b*) articles that were not interviewing the same pool of referrals or that used an external CHR− group of healthy controls; (*c*) articles with overlapping datasets; (*d*) articles not reporting on type of recruitment strategies (*e*) articles investigating unselected samples. Specifically, in case of multiple publications deriving from the same study population, we selected the articles reporting the largest data set. Literature search was summarized according to the PRISMA guidelines.^28^

### Recorded Variables

Data extraction was independently performed by 2 investigators (M.C., G.R.). The primary outcome variable was the pretest risk of developing psychosis) in help-seeking patients selected for CHR assessment (see below). The secondary outcome variable was type of recruitment strategies (see below).

Additional exploratory moderators tested in supplementary analyses included the source of referrals as previously operationalized (table 4 in Schultze-Lutter et al^18^): proportion of referrals initiated by the mental health services (eg, psychiatrists and psychologists working in private practice or hospital, psychosocial counseling services including. school and university counselors), proportion of referrals initiated by the education sector (eg, teachers or university lectures), proportion of referrals initiated by other sources (eg, GP, nonpsychiatric medical specialists other counseling and welfare services).^18^ Further moderators were: type of CHR interview, use of a screening questionnaire to pre-select potential CHR patients, year of publication, region in which the study was conducted (Europe vs other), prevailing health care system (national health insurance system according to Beveridge, social security system according to Bismarck or private insurance system), baseline characteristics of CHR samples (sample sizes, mean age and range, proportion of females), exposure to antipsychotics at baseline, CHR criteria used to assess outcome, and follow-up time.

### Statistical Analysis

The primary aim was to address the magnitude and consistency of pretest risk of psychosis in help-seeking patients selected for CHR assessment. Pretest risk of psychosis was calculated as defined below here in table 1, by dividing the total number of ICD/DSM transitions to psychosis at follow-up over the total number of patients seeking help at high-risk services at baseline: pretest risk of psychosis = transitions/(CHR+ and CHR−).

**Table 1.** CHR testing

		Psychosis Onset as Defined With the ICD/DSM Standard	
		Developing Psychosis	Not Developing Psychosis
CHR assessment outcome	*At Risk (CHR+*)	CHR+ transition	CHR+ nontransition
	*Not At Risk (CHR*−)	CHR− transition	CHR− nontransition

**Fig. 3.**
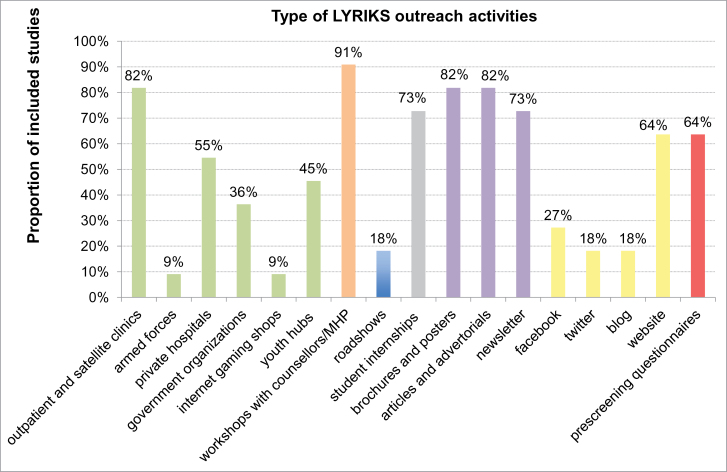
Type of Outreach LYRIKS activities adopted by each of the CHR studies included in the current database. Outreach activities were defined and clustered as indicated in the community engaged framework adopted by the LYRIKS CHR study.^15^ These included the following domains: (A) *screening assessments and recruitment, in green* (outpatient and satellite clinics, armed forces, private hospitals, government organizations, internet gaming shops, and youth hubs); (B) *workshops involving various community partners such as counsellors and mental healthcare professionals, in orange*; (C) *roadshows, in blue*; (D) *student internships, in gray*; (E) *print media, in violet* (brochures and posters, articles and advertorials, newsletter); (F) *social media, in yellow* (facebook, twitter, blog, website).^15^ The LYRIKS domains were further expanded with the addition of (G) *prescreening questionnaires, in red*. MHP, Mental health professionals.

The secondary aim was to investigate the impact of recruitment strategies on pretest risk of psychosis. Type of recruitment strategies was operationalized as: (*a*) main target of outreach campaign, (*b*) number of outreach activities conducted per study, (*c*) proportion of self-referrals. The main target of outreach campaign was clustered in 3 different outreach campaign approaches, on the basis of information collected from each study (detailed in table 2): mainly targeting mental health professionals and services, mainly targeting the general public, and mixed (ie, targeting both). The number of outreach activities per study was operationalized as in the LYRIKS study^29^ (and revised with the addition of prescreening questionnaires), and it was collected from the authors (see figure 3 for details). The proportion of self-referrals was assessed as the proportion of referrals initiated by self, family, friends.^18^

**Table 2.** Main target of outreach campaign

Study ID	**Description of Outreach Campaign (and Corresponding LYRIKS Domains**)	**Description of Source of Referrals**	**Main Target of Outreach Campaign**
1.Klosterkötter 2001^32 ^	No outreach campaign for the general public. Referrals invited “for diagnostic clarification of a possibly incipient schizophrenic disorder”.^32 ^ (LYRIKS none).	**“**Patients referred to outpatient departments of psychiatric university departments”^32 ^ because of “difficulties that had arisen in the diagnostic and therapeutic procedure”.^32 ^	Mainly targeting mental health professionals and services
2.Yung 2008^1 ^	Intensive outreach campaign to increase awareness of youth mental health and psychotic or prodromal symptoms in the general public. (LYRISK i–vi).	Referrals from a “range of sources including GPs and other primary care services, drug and alcohol services, school and university counselling services, and families/carers or young people themselves”.^1 ^ Some referrals (eg, to Youthscope (YS) were made “for help-seeking young people with nonpsychotic disorders”.^1 ^ “Most referrals were made by the participants themselves or their family members”.^11 ^	Mainly targeting the general public
3.Riecher-Rössler 2008^24 ^	“Regular information campaigns with scientific symposia and teaching courses for general practitioners, psychiatrists, social service staff”.^24 ^ Public alerted by “articles published in local newspapers and a special website”^24 ^ and by “a onepage checklist, to be used by potential referrers or lay people”.^24 ^ (LYRIKS i, ii, iv, v, vi, vii).	The main source of referrals was the local “Psychiatric Outpatient Department”^24 ^ and the Crisis Intervention Unit. However “other referrals come from relatives, or are self-referrals”.^24 ^	Mixed
4.Woods 2009^36 ^	Major involvement with mental heath services and professionals. Involvement of the general public with “distribution of educational brochures to gatekeepers, potential participants and their families and use of web resources”.^14 ^ Other general public initiatives included “academic detailing, grand rounds, educational talks, mailings, postings, websites and internet hits, and public service announcements”^19 ^ (from NAPLS-2, that explicitly attempted to replicate NAPLS-1). (LYRIKS i, ii, iv, v, vi, vii).	Multisite. In some sites the majority of subjects are usually referred by affiliated outpatient and inpatient psychiatry departments.^46 ^ In other sites “outpatient clinicians, a group consisting of outpatient psychiatrists, psychologists and mental healthcare practitioners, accounted for the majority of referrals”.^14 ^ In other sites the majority of the referrals were “self-referrals followed by family and friends”.^19 ^	Mixed
5.Addington 2012^47 ^	Service promotion included involvement of mental health agencies, private hospitals and government organizations as well as youth hubs, various general public partners such as counsellors and the use of brochures and posters, articles and advertorials, newsletter, website. (LYRIKS i, ii, iv, v, vi, vii).	Multisite. Most relevant source of referral were psychiatrists and psychologists working in private practice or public mental health hospitals, psychosocial counselling services including school and university counsellors. Self-referrals were also possible.	Mixed
6.Liu 2011^35 ^	Multifaceted information campaign targeting both general public-based populations, “which involved high school teachers, college and public counselling services, the high risk family”,^48 ^ and “mental health professionals, psychiatric clinics affiliated to the university hospital, general hospitals in metropolitan”.^48 ^ (LYRIKS i, ii, v, vii).	Referrals presenting with “CASIS”^48 ^ cognitive deficits, affective symptoms, social isolation, and school failure symptoms subsequently assessed in a special clinic focusing on “thought and perception disturbance”.^48 ^ Most referrals were coming from welfare services. To invite more subjects for assessment a low-threshold (worrying if at risk of psychosis)^48 ^ was applied.	Mixed
7.Simon 2012^49 ^	Service promotion included use of brochures and posters, articles and advertorials, newsletter, various general public partners such as counsellors and mental healthcare professionals. (LYRIKS i, ii, iv, v).	Patients mostly recruited from a clinical research facility which functions as an outpatient clinic. Self referrals also allowed.	Mixed
8.Lee 2013^29 ^	Extensive outreach general public campaign targeting “youth with a first-degree relative with psychosis; with deterioration in functioning; and/or those receiving help for nonspecific behavioural problems were targeted”.^15 ^ These included the involvement of armed forces, government organizations, internet gaming shops, youth hubs, various general public partners such as counsellors, roadshows, brochures and posters, articles and advertorials, newsletter, Facebook, twitter, blogs, websites.^15 ^ (LYRIKS i–vii).	“A public health initiative had resulted in collective support from the community partners in terms of subject recruitment.”^15 ^ Most referrals came from the general public.	Mainly targeting the general public
9.Schultze-Lutter 2014^50 ^	Information campaign “primarily aimed at mental health professionals as well as institutions and persons who might be contacted by help-seeking persons”^18 ^ and “on a smaller scale the general public was targeted”.^18 ^ (LYRIKS ii, iv, v, vi, vii).	“Mental health care professionals along with counselling services were the most valuable source of referrals”.^18 ^	Mainly targeting mental health professionals and services
10.Kotlicka-Antczak 2014^51 ^	Specific “training and workshops for psychiatrists and psychologists”^49 ^ along with “educational meetings and workshops for adolescents, teachers and parents in high schools”.^49 ^ Website. (LYRIKS i, ii, v, vi, vii).	“Referrals are accepted from outpatient and inpatient psychiatric centres”.^51 ^ Self-referrals from the general public by telephone or through the website.	Mixed
11.Spada 2015^52 ^	“Meetings of service promotion”^52 ^ for “Child and Adolescent neuropsychiatrists of the NHS, private practitioners, GPs, psychologists and psychotherapists”.^52 ^ Patients potentially eligible were “informed by their GPs or NHS’s CAMHS”.^52 ^ (LYRIKS i, ii, iv).	“The majority of the patients were referred by the inpatient unit of Child and Adolescent Neuropsychiatry”.^52 ^	Mainly targeting mental health professionals and services

Meta-analysis was conducted in “Metaprop” package of R 3.1.2 software (Comprehensive R Archive Network, http://cran.r-project.org/). This package is specifically developed to perform meta-analyses of proportions, implementing the DerSimonian-Laird method and logit transformations with a continuity correction for studies with a zero cell count. The influence of the type of recruitment strategies on pretest risk for psychosis (ie, main target of outreach campaign, number of outreach activities per study and proportion of self-referrals) was tested using meta-regression analyses. The slope of meta-regression line (β-coefficient: direct [+] or inverse [−]) indicates the strength of a relationship between moderator and outcome. Additional exploratory meta-regressions were conducted to test the secondary moderators listed above using Bonferroni’s correction for multiple testing.

Heterogeneity among study point estimates was assessed using Q statistics with the proportion of the total variability in the effect size estimates being evaluated with the *I* ^2^ index,^30^ which does not depend upon the number of studies included. As meta-analysis of observational studies is supposed to be characterized by significant heterogeneity, random effect models were used. Because conventional funnel plots used to assess for potential publication biases are inaccurate for meta-analyses of proportion studies with low proportion outcomes, we used a funnel plot of study size as recommended.^31^ Sensitivity analyses by removing studies one by one and rerunning the analysis were conducted to test robustness of results.

## Results

### Descriptive Characteristics of the Database

The literature review produced 11 independent studies that met the inclusion criteria (see PRISMA supplementary figure 1 and supplementary table 2), for a total of 2519 (CHR+: *n* = 1359; CHR−: *n* = 1160) help-seeking patients selected for CHR assessment. For excluded studies see supplementary table 3.

Four studies employed the Comprehensive Assessment of At-Risk Mental States (CAARMS), 3 the Structured Interview for Prodromal/Psychosis-Risk Symptoms (SIPS), 1 the Basel Screening Instrument for Psychosis (BSIP), 1 the Bonn Scale for the Assessment of Basic Symptoms (BSAPS), and 2 both the SIPS and Schizophrenia Proneness Instrument, Adult version (SPI-A). The mean follow-up time was 37.72 months (SD 27.81, median = 33) (supplementary table 2).

The type of the outreach LYRIKS activities and their main targets, as employed by the 11 included studies are qualitatively summarized in figure 3, and table 2, respectively. Overall, outreach campaigns mainly targeted mental health professionals and institutions, predominately by means of workshops with counsellors and mental health professionals, outpatients and satellite clinics and print media. Although general public-targeting activities and the use of social media were rare, most services would operate a website. The proportion of self-referrals across studies was on average only 22.07% (median = 21%, 95% CI: 7.65%–36.49%).

### Pretest Risk of Psychosis in Help-Seeking Patients Selected for CHR Assessment

**Fig. 4.**
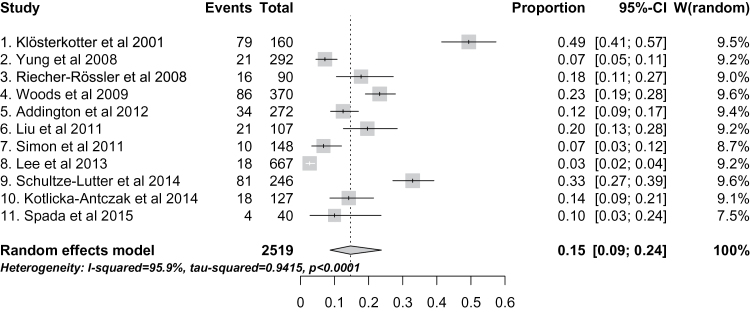
Meta-analytical estimate of pretest risk of developing psychosis (at an average follow-up of 38 months) in 2519 help-seekers at high-risk services reported in the 11 studies included. There was large and significant between-studies heterogeneity.

There was high heterogeneity across studies (*I* ^2^ = 96, *P* < .001), with high variability of pretest risk of psychosis at the average 38-month follow-up, ranging from 3%^29^ to 49%.^32^ The overall meta-analytical estimate of pretest psychosis risk in help-seeking patients selected for CHR assessment (both CHR+ and CHR−), mainly of high-risk services, is 15% (95% CI: 9%–24%) (figure 4). Sensitivity analyses confirmed robustness of the results. The funnel plot did not reveal significant publication biases (test for publication biases, *P* > .05).

### Impact of Type of Recruitment Strategies on Pretest Riks of Psychosis

**Fig. 5.**
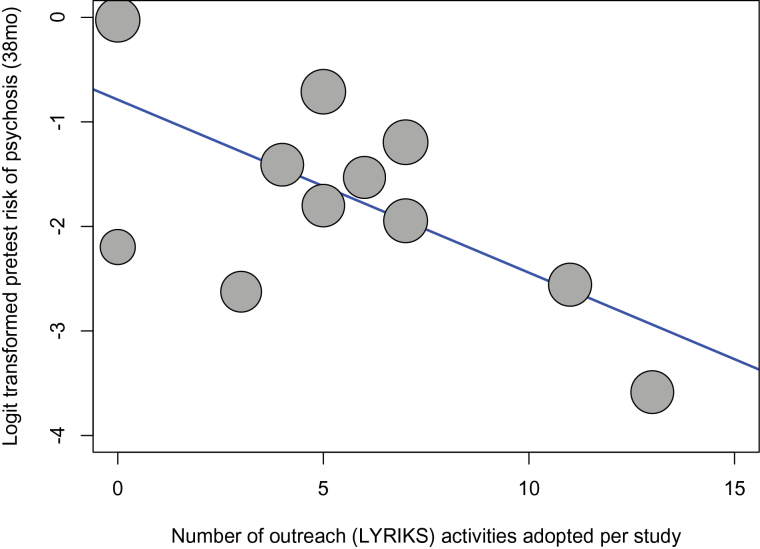
Metaregression of the number of outreach LYRIKS activities (see figure 3) adopted by each study and pretest psychosis risk (at an average follow-up of 38 months) in subjects recruited for CHR assessment.

There was a significant impact of type of recruitment strategies on the pretest psychosis risk of samples selected to undergo CHR assessment. Studies primarily directing their outreach campaigns to mental health services and with few self-referrals had increased pretest risk of psychosis compared to studies primarily reaching out to the general public and with a high proportion of self-referrals, with studies adopting a mixed outreach approach lying in an intermediate position (*n* = 11, β = −1.15, intercept = 0.491, *Z* = −4.620, *Q* = 21.35, *P* < .001, 95% CI: from −1.632 to −0.659, *R* ^2^ = 74.65). Intensive outreach campaigns dilute pretest risk of psychosis, as confirmed when the total number of LYRIKS outreach activities was used as moderator (*n* = 11, β = −.166, intercept = −0.788, *Z* = −3.073, *Q* = 9.441, *P* = .002, 95% CI: from −0.271 to −0.059, *R* ^2^ = 59.03, figure 5). Further, a higher proportion of self-referrals was inversely correlated with the pretest risk of psychosis (*n* = 10, β = −.029, intercept = −1.143, *Z* = −2.065, *Q* = 4.262, *P* = .039, 95% CI: −0.058 to −0.002, *R* ^2^ = 44.75). Supplementary meta-regressions did not reveal any additional significant moderator on pretest risk of psychosis (supplementary table 4).

## Discussion

This is the first meta-analysis to investigate the pretest risk of psychosis in different help-seeking patient samples selected to undergo a CHR interview as a function of their recruitment, ie, of their population of origin. We found an overall meta-analytical estimate of pretest psychosis risk of 15% over 38 months on average, with a high heterogeneity across studies. This heterogeneity was mostly explained by the type of recruitment strategies: intensive outreach campaigns primarily targeting the general public with a higher proportion of self-referrals were associated with dilution of pretest psychosis risk in help-seeking patients selected to undergo CHR assessment.

Our study provides the first meta-analytical evidence for risk enrichment in samples selected for CHR assessment. Indeed, the average 15% risk of pretest risk of psychosis in these help-seeking samples is significantly higher than the comparable 0.1% risk of psychosis in the general population over the same period of 38 months (incidence of psychosis 0.0317 per 100 person-years 95% CI: 0.025–0.041),^21^ and even higher than the 3.27% (95% CI 2.84–3.62)^33^ lifetime prevalence of nonorganic psychosis in the general population. Given that recruitment strategies significantly increase psychosis risk in help-seeking patients even before they undergo CHR assessment (pretest risk of psychosis), it is not only the criteria themselves that determine the posttest risk of transition to psychosis but also the process of preselection of samples, ie, the defined populations of origin of these samples, which creates substantial enrichment in risk.^34^ This was already recognized by the founders of the CHR criteria who noted that “the predictive power (ie, the positive and negative predictive value) of the CHR criteria is dependent on the population from which the sample is drawn”^1^(abstract). In discussion of current findings on the psychosis-predictive value of CHR criteria, typically there is little consideration of the differences in CHR recruitment across centers beyond their common focus on help-seekers and, consequently, of the associated pretest risk of psychosis being dependent from the adopted recruitment strategies.^34^ In other words: the pretest risk of psychosis in patients who seek help for mental problems and are selected for CHR assessment is often treated as being equal across studies. However, results of our meta-analysis show large heterogeneity, challenging the existence of a unitary pretest risk for psychosis across CHR studies. A good understanding of the pretest risk of psychosis of different populations is important for more accurately estimating a patient’s individual posttest risk of psychosis^7^ (see figure 1). Consequently, we further sought to address the moderators of heterogeneity in risk enrichment, focusing on numbers of outreach activities, main targets of outreach campaigns and proportion of self-referrals as proxy indexes of the type of recruitment strategies adopted.

With respect to the type and main target of outreach campaign employed to promote recruitment, some CHR studies showed an extensive use of multiple resources targeting the general public.^29^ Conversely, other CHR studies had no outreach campaign for the general public and were only^32^ or primarily^18^ targeting mental health professionals. Most studies adopted a mixed and multifaceted information campaign, targeting both general public samples such as high school teachers, college, and high-risk families as well as mental health professionals working in private practice or hospitals.^35,36^ Overall, a picture of CHR recruitment strategies following nonprobability sampling procedures that are opportunistic and poorly reproducible was clearly evident from figure 3 and table 2. Yet despite these shortcomings in comparable campaigns and in their description, our first meta-regression showed that campaigns primarily targeting the general public generally seem to be related to a lower pretest risk of psychosis as compared to campaigns mainly targeting mental health professionals and institutions. This was further supported by the second meta-regression showing that the overall intensity of outreach campaign (ie, number of LYRIKS activities per study, figure 5) diluted pretest risk of psychosis. Today, little is known about the prevalence and clinical significance^37,38^and even less about the psychosis-predictive ability of CHR criteria in the general population when assessed with any established psychometric CHR interview. The potential clinical insignificance of CHR symptoms and criteria in the general population,^7^ at least in younger age groups,^37^ warrants offering some caution against public campaigns that might unnecessarily unsettle persons with such potentially benign experiences.^39^ Thus, our finding of a lesser pretest risk of psychosis in CHR studies with intensive outreach campaigns targeting the general public seems to support the prevailing practice of concentrating outreach activities on mental health professionals and institutions.

These considerations are generally reinforced by our third meta-regression of the impact of self-referrals on the pretest risk of psychosis in help-seeking patients selected for CHR assessment. In line with the above findings, we found that higher proportions of self–referrals—a likely consequence of more intense campaigns targeting the general public—decreased the pretest risk of psychosis in the pool of CHR assessed patients. In line with this interpretation, the CHR study with the highest pretest risk enrichment (psychosis risk = 0.49 by almost 10 years),^32^ had also the highest threshold for referrals, exclusively from mental health professionals/services, as there was no formal high-risk service set up and no outreach campaign carried out. Thus, this study adopted selective judgmental and deviant sampling techniques with no self-referrals.^32^ Before their selection for psychosis-risk assessment, “all patients had sought help for various complaints from various clinicians, and the referrals were because of difficulties that had arisen in the diagnostic and therapeutic procedure”.^32^ This indicates recruitment of a sample which had become increasingly enriched with psychosis-prone patients while passing through several mental health service filters. Conversely, the study with the lowest pretest risk enrichment (psychosis risk = 0.035 at more than 30 months) had adopted an extensive outreach campaign targeting the general public, designed as a public health initiative and resulting in a large proportion of self-referrals.^29^

Furthermore, our meta-analytical results are in line with the pioneering study exploring the impact of 2 types of recruitment strategies on the CHR state: a screening method in a consecutive help-seeking population entering secondary mental health services vs a less systematically selected sample referred to a high-risk service.^20^ The authors found that the screening in secondary mental health services was associated with a higher risk of transition to psychosis within 12 months.^20^ Our results are thus providing meta-analytical support to the hypothesis that increased awareness of youth mental health and psychotic or CHR symptoms in the general population and, consequently, more self-referrals contributes to the global decline in transition risks.^11^ Our results are in line with the explanation that this is due to the inclusion of more false positives because of “altered referral patterns”.^11^ Vice versa, the explanation of declining short-term transition risks because of earlier referral when psychosis onset is less imminent^11^ seems not to be supported, as the steady long-term increase in transition rates that would be expected from this explanation was not observed by recent meta-analyses.^9,40^ Our findings fit well with a differential propensity in recognition of putative CHR symptoms across different referral sources, explaining our meta-analytical impact of the proportion of self-referrals on the psychosis risk enrichment. Patients, family members and friends all usually realize that “something is not quite right” mostly with the person’s psychological and social functioning^13^ and thus may be inclined to encourage early help-seeking even for nonspecific symptoms at a service with a low threshold for entry and well-known for its intensive outreach campaign. Conversely, mental health professionals will have become more aware of CHR symptoms and thus more likely to detect and refer true CHR cases. Interestingly, a study of the differential propensity in recognition of CHR symptoms between different referrers concluded that psychiatrists were most likely to consider schizophrenia for all stages of clinical severity, whereas school counselors were least likely to.^41^ Thus, where outreach campaigns are less directed to the general public with less increase of self-referrals, by the time potential CHR patients are filtered by mental health services, CHR symptoms may have become long-lasting and more prominent and functionally impairing so that transition becomes more likely.^11^ Indeed, there is evidence that most people who develop a psychotic disorder sought help in secondary mental health services prior to the onset of psychosis.^42^ Of interest, our metaregression analyses found no age effect on pretest risk of psychosis, due to the limited number of studies and reduced variability of this moderator. Two studies^12,37^ and meta-analyses^9,40^ have indicated a lower risk of psychosis in children and young adolescent populations that are increasingly targeted by high-risk services. Thus increasing recruitment from this young age group might further contribute to the apparent risk dilution.^12^

Overall, our findings highlight a dilemma that CHR researchers and clinicians are facing: identifying and treating CHR patients either too late (eg, after passing through several professional filters) or too early (eg, self-referrals after intensive awareness programs) and thus being too exclusive or too inclusive.^43^ On the one hand, intensive outreach campaigns targeting the general public and resulting in high numbers of self-referrals dilute the risk enrichment of samples selected for CHR assessment and their pretest risk of psychosis onset, reducing by consequence the positive predictive value of CHR criteria and leading to the initiation of preventative clinical interventions in persons who do not need them. On the other hand, purposive and deviant sampling by mental health professionals of patients filtered through mental health services can select patient samples with a high enrichment of pretest psychosis risk, at the expense of possibly missing early cases from the general population. There is no simple solution to such a dilemma. One possibility to move beyond purposive sampling would be to adopt a sequential monitoring procedure^20^ and to experimentally monitor risk enrichment across its sequential steps. However, it may be more relevant to better investigate the cumulative impact of different sociodemographic, environmental, clinical, neurobiological risk factors on the pretest risk of psychosis of populations from which samples are recruited for CHR assessment, and thus to finally be able to more exactly estimate a person’s posttest risk for psychosis. Finally, our results suggest that there is a wealth of clinical knowledge which is relevant for high-risk services but is not coded in the international CHR manuals. Future CHR studies should clearly describe their recruitment strategies, including type and targets of outreach campaign and source of referrals.

## Conclusions

Recruitment strategies still represent the dark side of the moon in CHR research. They lead to significant psychosis risk enrichment: from 0.1% pretest risk of psychosis (at 38 months) in the general population to an average risk of 15% (at 38 months) in samples recruited for CHR assessment. Yet despite their significance, recruitment strategies are usually underreported, poorly detailed, opportunistic and nonstandardized, producing high heterogeneity of posttest probability across CHR studies. However, our meta-analysis clearly indicated that intensive outreach information campaigns predominantly targeting the general public and higher proportions of self-referrals dilute the psychosis risk in the samples selected for CHR assessment, despite the likely welcomed effect of bringing persons with mental problems earlier into contact with a mental health service. Thus, recruitment of CHR patients remains challenging for the ongoing and unresolved clinical dilemma of identifying them either too late or too early.

## Supplementary Material

Supplementary material is available at http://schizophreniabulletin.oxfordjournals.org.

## Funding

P.F-P. was supported in part by a 2014 NARSAD Young Investigator Award. Some data in this study was supported by the following NIMH grants U01MH06634 to J.A., U01MH0660694 to D.P., U01MH066160 to S.W.

## Supplementary Material

Supplementary Data
