# Supplementary material for: The Dark Side of the Moon: Meta-analytical Impact of Recruitment Strategies on Risk Enrichment in the Clinical High Risk State for Psychosis
Source: Schizophr Bull. 2015 Nov 20;42(3):732–43. doi: 10.1093/schbul/sbv162 (PMC4838090; doi:10.1093/schbul/sbv162)
Supplement: Supplementary Data [file supp_42_3_732__index.html]

The Dark Side of the Moon: Meta-analytical Impact of Recruitment Strategies on Risk Enrichment in the Clinical High Risk State for Psychosis — The Dark Side of the Moon: Meta-analytical Impact of Recruitment Strategies on Risk Enrichment in the Clinical High Risk State for Psychosis — Supplementary Data 

# The Dark Side of the Moon: Meta-analytical Impact of Recruitment Strategies on Risk Enrichment in the Clinical High Risk State for Psychosis

## Supplementary Data

Data files

- Supplementary Data - Supplementary Data
