# Supplementary material for: The Dark Side of the Moon: Meta-analytical Impact of Recruitment Strategies on Risk Enrichment in the Clinical High Risk State for Psychosis
Source: Schizophr Bull. 2015 Nov 20;42(3):732–43. doi: 10.1093/schbul/sbv162 (PMC4838090; doi:10.1093/schbul/sbv162)
Supplement: Supplementary Data [file supp_sbv162_Recruitment_CHR_Suppl_R_Sep29_CLEAN.doc]

**Supplementary Material to:**

**THE DARK SIDE OF THE MOON:**

**META-ANALYTICAL IMPACT OF RECRUITMENT STRATEGIES ON THE CLINICAL HIGH RISK STATE FOR PSYCHOSIS**

**by:**

Fusar-Poli P, Schultze-Lutter F, Cappucciati M, Rutigliano G, Bonoldi I, Stahl D, Borgwardt S, Riecher-Rossler A, Addington J, Perkins D, Woods SW, McGlashan TH, Lee J, Klosterkötter J, Yung AR, McGuire P.

**Supplementary S1**. Psychometric CHR interviews

The established CHR psychometric interviews currently used include the Comprehensive Assessment of At Risk Mental State (CAARMS)^1^, the Structured Interview for Psychosis-Risk Syndrome (SIPS)^2^, the Basel Screening Instrument for Psychosis (BSIP)^3^, the Bonn Scale for the Assessment of Basic Symptoms (BSABS)^4^ and its successor instruments, the Schizophrenia Proneness Instrument, Adult (SPI-A^5^), and Child & Youth version (SPI-CY^6^). These interviews assess the ‘ultra-high risk’ (UHR) criteria ^1^ (i.e., attenuated psychotic symptoms (APS), brief limited intermittent psychotic symptoms (BLIPS) and genetic trait vulnerability plus a marked decline in psychosocial functioning (GRFD)) and the basic symptom (BS) criteria ^7^ (i.e., cognitive disturbances (COGDIS) and cognitive-perceptive BS (COPER)).

**eTable 1.** *Moose checklist.*

| **Criteria** | | **Brief description of how the criteria were handled in the meta-analysis** |
| --- | --- | --- |
| **Reporting of background should include** | |  |
| √ | Problem definition | The individual probability of developing psychosis after being tested positive or negative for clinical high risk (CHR) criteria (posttest probability) is dependent on the pre-test probability of the population from which the person is selected, and thus on recruitment. Yet, the impact of recruitment strategies on pretest probability is unknown. |
| √ | Hypothesis statement | We tested the pre-test probability in patients selected for CHR assessment. We further tested the impact of recruitment strategies on pre-test probability. |
| √ | Description of study outcomes | Main outcome  Magnitude and consistency of pretest probability in help seeking patients selected for CHR assessment:  Secondary outcome  Impact of type of recruitment strategies on pretest probability |
| √ | Type of exposure or intervention used | Recruitment strategies employed to select patients undergoing CHR assessment: main target of outreach campaign, number of outreach activities per study and proportion of self-referrals |
| √ | Type of study designs used | Longitudinal studies |
| √ | Study population | Help-seeking patients selected for CHR assessment |
| **Reporting of search strategy should include** | |  |
| √ | Qualifications of searchers | The credentials of the investigators are indicated in the author list and in the acknowledgements. |
| √ | Search strategy, including time period included in the synthesis and keywords | The search was extended until March 2015, and included abstracts in English and German language only. The electronic research adopted several combinations of the following keywords: The electronic research adopted several combinations of the following keywords: “at risk mental state”, “psychosis risk”, “prodrome”, “prodromal psychosis”, “ultra-high risk”, “high risk”, “help-seeking”, “referral”, “recruitment”, “psychosis prediction”, “psychosis onset” and names of possible CHR instruments A second step involved the use of Scopus® and a manual search of the reference lists of the retrieved articles. |
| √ | Databases and registries searched | Web of Science^SM^, MEDLINE® and Scopus®. |
| √ | Search software used, name and version, including special features | Web of Knowledge^SM^ and Scopus®. |
| √ | Use of hand searching | We hand-searched bibliographies of retrieved papers for additional references. |
| √ | List of citations located and those excluded, including justifications | Details of the literature search process are outlined in the supplementary materials. |
| √ | Method of addressing articles published in languages other than English | The search included abstract in English and German language only. We were able to read those abstract written in German language. |
| √ | Method of handling abstracts and unpublished studies | Abstracts and unpublished studies were excluded. |
| √ | Description of any contact with authors | We contacted all the corresponding authors to provide additional data when needed. We developed a specific checklist to collect data on source of referrals, type and target of outreach campaign, on the basis of previous studies. |
| **Reporting of methods should include** | |  |
| √ | Description of relevance or appropriateness of studies assembled for assessing the hypothesis to be tested | Detailed inclusion and exclusion criteria were described in the methods section. |
| √ | Rationale for the selection and coding of data | Data extracted from each of the studies were relevant to the population characteristics, study design, exposure, outcome, and possible effect of confounders. |
| √ | Assessment of confounding | Several meta-regressions were used to examine the influence the a-priori moderator and of additional supplementary moderators (correcting for multiple comparisons) |
| √ | Assessment of study quality, including blinding of quality assessors; stratification or regression on possible predictors of study results | Not conducted, we used sample size as proxy measure of quality of studies. |
| √ | Assessment of heterogeneity | Heterogeneity across studies was assessed using the I^2^, with values of 25%, 50%, and 75% representing mild, moderate, and severe inconsistency, respectively. The meta-regression were additionally planned to address unexplained heterogeneity |
| √ | Description of statistical methods in sufficient detail to be replicated | Description of methods of meta-analyses, sensitivity analyses, meta-regression and assessment of publication bias are detailed in the methods. |
| √ | Provision of appropriate tables and graphics | We included the PRISMA flow-chart and several tables to describe the literature search and its results. Several graphs were used to describe the main findings of the analyses. |
| **Reporting of results should include** | |  |
| √ | Graph summarizing individual study estimates and overall estimate | We have appended them in the main text. Additional graphs were presented as supplementary material to fully describe the results. |
| √ | Table giving descriptive information for each study included | Results appended in specific tables are recommended |
| √ | Results of sensitivity testing | Sensitivity analyses (i.e. exclusion of 1 study at a time) were conducted and findings reported in the main text |
| √ | Indication of statistical uncertainty of findings | 95% confidence intervals used consistently across the analyses |
| **Reporting of discussion should include** | |  |
| √ | Quantitative assessment of bias | Performed as indicated in the methods with a funnel plot of study size |
| √ | Justification for exclusion | Exclusion criteria were: (a) abstracts, pilot datasets, and paper in languages other than those above; (b) articles that were not interviewing the same pool of referrals or that used an external CHR- group of healthy controls; (c) articles with overlapping datasets; (d) articles investigating unselected samples. These exclusion criteria were needed to investigate pre-test probability in help seeking patients selected for CHR assessment. |
| √ | Assessment of quality of included studies | We have used sample size as a proxy of quality of study in meta-regression analyses. |
| **Reporting of conclusions should include** | |  |
| √ | Consideration of alternative explanations for observed results | We discussed alternative explanations for our findings, specifically considering potential methodological shortcomings. |
| √ | Generalization of the conclusions | We presented estimates based on 95% CI of meta-analytical pre-test probability in CHR patients. The results were appended in the main text and in the supplementary material. |
| √ | Guidelines for future research | We have addressed them in the discussion suggesting specific streams of future investigations and recommending detailed reporting of recruitment strategies in CHR studies |
| √ | Disclosure of funding source | None |


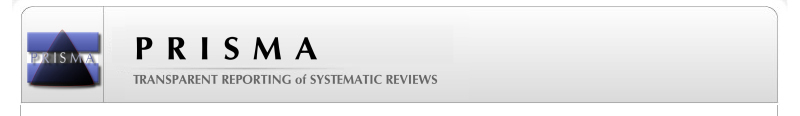
**eFigure 1.** *PRISMA Flow Chart*

**Identification**

**Eligibility**

**Included**

**Screening**

Full-text articles (PDFs) excluded
(n = 108):

Overlapping dataset: (n= 5)

No HR+ group: (= 2)

No HR- group: (= 87)

HR- not followed-up: (= 8)

HR+ not followed-up: (=4)

HR- non help-seeking (n=2)

Abstracts identified through manual search (n = 96)

Full-text articles (PDFs) assessed for eligibility
(n = 118)

Abstracts excluded on initial review (n = 1254)

Abstracts screened
(n = 1372)

Abstracts after duplicates removed
(n = 1372)

Abstracts identified through database searching (Web of Knowledge^SM^) (n = 1388)

Studies included in the meta-analysis
(n = 10 *) for a total of 11 datasets

* One study provided two independent datasets

**eTable 2**. *Independent studies included in the meta-analysis (studies n=11, subjects n=2519; CHR+: n=1359, CHR-: n= 1160)*

| Study | Exposure to antipsychotics at baseline | Predictor  (Index test) | Psychosis diagnosis  (Reference standard) | Age  (mean±SD, range) | Gender  (% females) | Follow-up  (months) | CHR+  (baseline) | CHR-(baseline) |
| --- | --- | --- | --- | --- | --- | --- | --- | --- |
| 1. Klosterkötter, et al. 2001^8^ (a) | NO | BSABS (BS) | DSM-IV | 29.3±10.0  (15-53) | 47.5 | ≥30 | 110 | 50 |
| 2. Yung, et al. 2008^9^ | YES (Na) | CAARMS, before 2006 (UHR) | CAARMS | 18.1  (15-24) | 51.0 | 6 (b), 24 | 119 | 173 |
| 3. Riecher-Rössler, et al. 2008^3^ | NO | BSIP (UHR plus 4^th^ criterion) | BPRS | 26.8±8.9  (18-60) | 41.4(c) | 6, 12, 24, ≥30 | 58 | 32 |
| 4. Woods, et al. 2009 ^10^(d) | YES (11.6%) | SIPS (UHR) | DSM-IV or medical records | 17.8±4.4  (12-36) | 39.5 | 6, 12, 24 | 259 | 111 |
| 5. Addington, et al. 2012 ^11^(d) | YES (1.8%) | SIPS (UHR) | DSM-IV | 19.76±4.5  (12-31) | 47.8 | 6, 12, 24 | 172 | 100 |
| 6. Liu, et al. 2011^12^ | YES (79.7%)(c) | SIPS (UHR) | DSM-IV | 21.4±4.0  (16-24) | 47.7 | 24 | 59 | 48 |
| 7. Simon, et al. 2012^13^ | NO | SIPS / SPI-A (e) (BS / UHR) | DSM-IV | 21.0  (14-40) | 32.4 | 12, 24 | 99 | 49 |
| 8. Lee, et al. 2013^14^(d) | NO | CAARMS before 2006 (UHR) | DSM-IV | 21.6±3.5  (14-29) | 39.9 | 0, 6(c), 12, 24, ≥30 | 173 | 494 |
| 9. Schultze-Lutter, et al. 2014^15^ | YES (13.8%) | SPI-A / SIPS (e) (BS / UHR) | DSM-IV | 24.9±6.0  (15-39) | 37.0 | 6, 12, 24, ≥30 | 194 | 52 |
| 10. Kotlicka-Antczak et al 2014^16^(d) | YES (10.2%) | CAARMS (UHR) | ICD-10 | 19.05±3.6  (15-29) | 51.1 (c) | ≥30 | 94 | 33 |
| 11. Spada, et al. 2015^17^ | NO | CAARMS (UHR) | DSM-IV | 15.8±1.7  (12-17) | 47.5 | 6 | 22 | 18 |
| UHR, Ultra High Risk; BS, Basic Symptoms, BSABS, Bonn Scale for the Assessment of Basic Symptoms; BPRS, Brief Psychiatric Rating Scale; BSIP, Basel Screening Instrument for Psychosis; CAARMS, Comprehensive Assessment of At Risk Mental State; DSM-IV, Diagnostic and Statistical Manual of Mental Disorders; SIPS, Structured Interview for Prodromal Syndromes; SPI-A, Schizophrenia Proneness Instrument; Na, not available; a) at least 1 BS; b) 6 months data reported in Yung et al 2006^18^; c) CHR+ only; d) updated follow-up data provided by the authors; e) different combinations of SPI-A and SIPS criteria reported in this article; NAPLS-1 data were used breaking up PREDICT data so the two samples are not overlapping. | | | | | | | | |

**eTable 3.** List of excluded studies and reason for exclusion

| **Study name and year of publication** | **Reason for exclusion** |
| --- | --- |
| T.J. Miller, et al. 2002^19^ | Overlapping sample with Woods, et al. 2009 |
| P. Miller, et al. 2002^20^ | No CHR sample |
| Phillips, et al. 2002^21^ | No CHR- group followed up |
| Miller, et al. 2003^22^ | Overlapping sample with Woods, et al. 2009 |
| Yung, et al. 2003^23^ | No follow-up of CHR- group |
| Brewer, et al. 2003^24^ | No CHR- group |
| Lencz, et al. 2003^25^ | No CHR- group |
| Woods, et al. 2003^26^ | No CHR- group |
| Yung, et al. 2004^27^ | No follow-up of CHR- group |
| Mason, et al. 2004^28^ | No CHR- group |
| Häfner, et al. 2004^29^ | No CHR- group |
| Yung, et al. 2005^30^ | Overlapping sample with Yung, et al. 2008 |
| Brewer, et al. 2005^31^ | No CHR- group |
| Francey, et al. 2005^32^ | No CHR- group |
| Yun, et al. 2005^33^ | No CHR- group |
| Garner, et al. 2005^34^ | No CHR- group |
| Job, et al. 2006^35^ | No CHR sample |
| Lam, et al. 2006^36^ | No CHR- group |
| Lemos, et al. 2006^37^ | No CHR- group |
| Keefe, et al. 2006^38^ | No CHR- group |
| Lencz, et al. 2006^39^ | No CHR- group |
| Velakoulis, et al. 2006^40^ | No CHR- group |
| Simon, et al. 2006^41^ | No follow-up of CHR sample |
| Haroun, et al. 2006^42^ | No CHR- group |
| Broome, et al. 2007^43^ | No CHR- group |
| Hoffman, et al. 2007^44^ | No CHR- group |
| Kristensen, et al. 2007^45^ | No CHR- group |
| Thompson, et al. 2007^46^ | No CHR- group |
| Pukrop, et al. 2007^47^ | No CHR- group |
| Broome, et al. 2007^48^ | No CHR- group |
| Nieman, et al. 2007^49^ | No CHR- group |
| Amminger, et al. 2008^50^ | No CHR- group |
| Borgwardt, et al. 2008^51^ | No CHR- group |
| Brockhaus-Dumke, et al. 2008^52^ | No CHR- group |
| Cannon, et al. 2008^53^ | No CHR- group |
| Walder, et al. 2008^54^ | No CHR- group |
| Walterfang, et al. 2008^55^ | No CHR- group |
| Hurlemann, et al. 2008^56^ | No CHR- group |
| Takahashi, et al. 2008^57^ | No CHR- group |
| Kéri, et al. 2009^58^ | No CHR- group |
| Koutsouleris, et al. 2009^59^ | No CHR- group |
| Koutsouleris, et al. 2009^60^ | No CHR- group |
| Lemos-Giraldez, et al. 2009^61^ | No CHR- group |
| Mittal, et al. 2009^62^ | No CHR- group |
| Riecher-Rossler, et al. 2009^63^ | No CHR- group |
| Salokangas, et al. 2009^64^ | No follow-up of CHR- group |
| Sun, et al. 2009^65^ | No follow-up of CHR- group |
| Velthorst, et al. 2009^66^ | No CHR- group |
| Myiakoshi, et al. 2009^67^ | No follow-up of CHR- group |
| Takahashi, et al. 2009^68^ | No CHR- group |
| Fusar-Poli, et al. 2009^69^ | No CHR- group |
| Simon, et al. 2010^70^ | No CHR- group |
| Bloemen, et al. 2010^71^ | No CHR- group |
| Bechdolf, et al. 2010^72^ | No CHR- group |
| Fusar-Poli, et al. 2010^73^ | No CHR- group |
| Korver, et al. 2010^74^ | No CHR- group |
| Mittal, et al. 2010^75^ | No CHR- group |
| Mossner, et al. 2010^76^ | No CHR- group |
| Nelson, et al. 2010^77^ | No CHR- group |
| Olvet, et al. 2010^78^ | No CHR- group |
| Ruhrmann, et al. 2010^79^ | No CHR- group |
| Schlosser, et al. 2010^80^ | No CHR- group |
| Jung, et al. 2010^81^ | No CHR- group |
| Sabb, et al. 2010^82^ | No CHR- group |
| Seidman, et al. 2010^83^ | No CHR- group |
| Ziermans, et al. 2011^84^ | No CHR- group |
| Bearden, et al. 2011^85^ | No CHR- group |
| Dragt, et al. 2011^86^ | No follow-up of CHR- group |
| Fontenelle, et al. 2011^87^ | No CHR- group |
| Raballo, et al. 2011^88^ | No CHR- group |
| Simenova, et al. 2011^89^ | No CHR- group |
| Thompson, et al. 2011^90^ | No CHR- group |
| Velthorst, et al. 2011^91^ | No CHR- group |
| Kim, et al. 2011^92^ | No CHR- group |
| Schimmelmann, et al. 2011^93^ | No follow-up of CHR sample |
| Addington, et al. 2011^94^ | No CHR- group |
| Demjaha, et al. 2012^95^ | No CHR- group |
| Dragt, et al. 2012^96^ | No CHR- group |
| Fusar-Poli, et al. 2012^97^ | No CHR- group |
| Koutsouleris, et al. 2012^98^ | No CHR- group |
| Schlosser, et al. 2012^99^ | No CHR- group |
| Bechdolf, et al. 2012^100^ | No CHR- group |
| Carriòn, et al. 2013^101^ | No CHR- group |
| Fusar-Poli, et al. 2013^102^ | No CHR- group |
| Nelson, et al. 2013^103^ | No CHR- group |
| Nieman, et al. 2013^104^ | No CHR- group |
| Nieman, et al. 2013^105^ | No CHR- group |
| Tarbox, et al. 2013^106^ | No HR- group |
| Chung, et al. 2013^107^ | No follow-up of CHR sample |
| Buchy, et al. 2014^108^ | No CHR- group |
| DeVylder, et al. 2014^109^ | No CHR- group |
| Manninen, et al. 2014^110^ | No help-seeking sample |
| Tay, et al. 2014^111^ | No CHR- group followed-up |
| Katsura, et al. 2014^112^ | No CHR- group |
| Koutsouleris, et al. 2014^113^ | No CHR- group |
| Lindgren, et al. 2014^114^ | Unselected help-seeking population, missing data |
| Modinos, et al. 2014^115^ | No CHR- group |
| Theodoridou, et al. 2014^116^ | No CHR- group |
| Thompson, et al. 2014^117^ | No CHR- group |
| Van Tricht, et al. 2014^118^ | No CHR- group |
| Chen, et al. 2014^119^ | No CHR- group |
| Braham, et al. 2014^120^ | No follow-up of CHR sample |
| Michel, et al. 2014^121^ | Overlapping sample with Schultze-Lutter, et al. 2014 |
| Woods, et al. 2014^122^ | Overlapping sample with Woods, et al. 2009 |
| Ramyead, et al. 2014^123^ | No CHR- group |
| Perkins, et al. 2014^124^ | No CHR- group |
| Ziermans, et al. 2014^125^ | No CHR- group |
| Poe, et al. 2014^126^ | No CHR- group |

**eTable 4**. Supplementary meta-regression analyses

| **Covariate** | **n** | **Beta** | **Intercept** | **Z** | **Q** | **P*** | **95%CI** | |
| --- | --- | --- | --- | --- | --- | --- | --- | --- |
| Follow-up (in months) | 11 | 0.025 | -2.732 | -1.732 | 2.9283 | ns | -0.008 | 0.0417 |
| Age (in years) | 11 | 0.135 | -4.664 | 1.946 | 3.787 | ns | -0.001 | 0.271 |
| Gender (% females) | 11 | 0.0158 | -2.453 | 0.286 | 0.082 | ns | -0.092 | 0.124 |
| Sample size (n) | 11 | -0.0027 | -1.122 | -1.623 | 2.639 | ns | -0.006 | 0.001 |
| Baseline use of antipsychotics (by at least some patients/by none) | 11 | 0.3725 | -1.970 | 0.5713 | 0.326 | ns | -0.905 | 1.650 |
| CHR instrument | 11 | -0.155 | -1.436 | -0.3986 | 0.159 | ns | -0.916 | 0.612 |
| Region (Europe vs other) | 11 | -0.699 | -0.731 | -1.229 | 1.510 | ns | -1.814 | 0.416 |
| Publication year | 11 | -0.124 | 246.59 | -1.593 | 2.537 | ns | -0.276 | 0.029 |
| Other referrals ^a^ | 10 | -0.0049 | -1.7808 | -0.1659 | 0.0275 | ns | -0.0633 | 0.0534 |
| Referrals from education sector ^b^ | 10 | -0.0632 | -1.5859 | -0.8036 | 0.6457 | ns | -0.2173 | 0.0909 |
| Mental health service referrals ^c^ | 10 | 0.0169 | -2.8785 | 1.7059 | 2.91 | ns | -0.0025 | 0.0362 |
| Type of local healthcare system ^d^ | 11 | -0.4071 | -0.8162 | -1.229 | 2.410 | ns | -0.9211 | 0.1068 |
| Pre-selection by a screening instrument (yes/no) | 11 | -0.2936 | -1.5409 | -0.4273 | 0.1826 | ns | -1.640 | 1.053 |

* p values adjusted for multiple testing

^a^ Includes referrals initiated by other sources: GP, non psychiatric medical specialists other counseling and welfare services

^b^ Includes referrals initiated by educational sources: psychiatrists and psychologists working in private practice or hospital, psychosocial counseling services incl. school and university counselors

^c^ Includes referrals initiated by the mental health services

^d^ The following health care models are followed in the countries in that the 11 studies included in the meta-analysis were conducted: Beveridge model in Canada, UK, Taiwan and Italy; Bismarck model in Germany and Switzerland; Beveridge model with elements of Bismarck in Singapore and Australia; the private insurance model in the US America,

**REFERENCES**

**1.** Yung AR, Yuen HP, McGorry PD, et al. Mapping the onset of psychosis: the Comprehensive Assessment of At-Risk Mental States. *Australian & New Zealand Journal of Psychiatry* 2005;39(11-12):964-971.

**2.** McGlashan T, Walsh B, Woods S. *The Psychosis-Risk Syndrome: Handbook for Diagnosis and Follow-Up*: Oxford Univ. Press; 2010.

**3.** Riecher-Rössler A, Aston J, Ventura J, Merlo M, Borgwardt S, Gschwandtner U, Stieglitz RD. The Basel screening instrument for psychosis (BSIP): Development, structure, reliability and validity. *Fortschr Neurol Psyc* Apr 2008;76(4):207-216.

**4.** Gross G, Huber G, Klosterkötter J, Linz M. *Bonner Skala für die Beurteilung von Basissymptomen. BSABS; Bonn Scale for the assessment of basic symptoms*. Berlin: Springer-Verlag; 1987.

**5.** Schultze-Lutter F, Addington J, Ruhrmann S, Klosterkötter J. *Schizophrenia Proneness Instrument, Adult version (SPI-A)*. Rome: Giovanni Fiorito Editore; 2007.

**6.** Schultze-Lutter F, Koch E. *Schizophrenia Proneness Instrument, Child and Youth version (SPI-CY)*. Rome: Giovanni Fioriti Editore s.r.l.; 2010.

**7.** Klösterkötter J GG, Huber G, Wieneke A, Steinmeyer EM, Schultze-Lutter F Evaluation of the Bonn Scale for the Assessment of Basic Symptoms - BSABS as an instrument for the assessment of schizophrenia proneness: A review of recent findings. *Neurology, Psychiatry and Brain Research* 1997;5:137-150.

**8.** Klösterkotter J, Hellmich M, Steinmeyer EM, Schultze-Lutter F. Diagnosing schizophrenia in the initial prodromal phase. *Archives of general psychiatry* Feb 2001;58(2):158-164.

**9.** Yung AR, Nelson B, Stanford C, et al. Validation of "prodromal" criteria to detect individuals at ultra high risk of psychosis: 2 year follow-up. *Schizophrenia research* Oct 2008;105(1-3):10-17.

**10.** Woods SW, Addington J, Cadenhead KS, et al. Validity of the prodromal risk syndrome for first psychosis: findings from the North American Prodrome Longitudinal Study. *Schizophrenia bulletin* Sep 2009;35(5):894-908.

**11.** Addington J, Piskulic D, Perkins D, Woods SW, Liu L, Penn DL. Affect recognition in people at clinical high risk of psychosis. *Schizophrenia research* Sep 2012;140(1-3):87-92.

**12.** Liu CC, Lai MC, Liu CM, et al. Follow-up of subjects with suspected pre-psychotic state in Taiwan. *Schizophrenia research* Mar 2011;126(1-3):65-70.

**13.** Simon AE, Gradel M, Cattapan-Ludewig K, Gruber K, Ballinari P, Roth B, Umbricht D. Cognitive functioning in at-risk mental states for psychosis and 2-year clinical outcome. *Schizophrenia research* Dec 2012;142(1-3):108-115.

**14.** Lee J, Rekhi G, Mitter N, et al. The Longitudinal Youth at Risk Study (LYRIKS)--an Asian UHR perspective. *Schizophrenia research* Dec 2013;151(1-3):279-283.

**15.** Schultze-Lutter F, Klosterkotter J, Ruhrmann S. Improving the clinical prediction of psychosis by combining ultra-high risk criteria and cognitive basic symptoms. *Schizophrenia research* Apr 2014;154(1-3):100-106.

**16.** Kotlicka-Antczak M, Pawelczyk T, Rabe-Jablonska J, Pawelczyk A. PORT (Programme of Recognition and Therapy): the first Polish recognition and treatment programme for patients with an at-risk mental state. *Early Interv Psychia* Apr 11 2014.

**17.** Spada G, Molteni S, Pistone C, Chiappedi M, McGuire P, Fusar-Poli P, Ballottin U. Identifying children and adolescents at ultra high risk for psychosis in Italian Neuropsychiatry Services: a feasibility study. *Europ Child and Adolesc Psychiatry in press* 2015.

**18.** Yung AR, Stanford C, Cosgrave E, Killackey E, Phillips L, Nelson B, McGorry PD. Testing the Ultra High Risk (prodromal) criteria for the prediction of psychosis in a clinical sample of young people. *Schizophrenia research* May 2006;84(1):57-66.

**19.** Miller TJ, McGlashan TH, Rosen JL, Somjee L, Markovich PJ, Stein K, Woods SW. Prospective diagnosis of the initial prodrome for schizophrenia based on the Structured Interview for Prodromal Syndromes: preliminary evidence of interrater reliability and predictive validity. *The American journal of psychiatry* May 2002;159(5):863-865.

**20.** Miller PM, Byrne M, Hodges A, Lawrie SM, Johnstone EC. Childhood behaviour, psychotic symptoms and psychosis onset in young people at high risk of schizophrenia: early findings from the edinburgh high risk study. *Psychological medicine* Jan 2002;32(1):173-179.

**21.** Phillips LJ, Curry C, Yung AR, Yuen HP, Adlard S, McGorry PD. Cannabis use is not associated with the development of psychosis in an 'ultra' high-risk group. *The Australian and New Zealand journal of psychiatry* Dec 2002;36(6):800-806.

**22.** Miller TJ, McGlashan TH, Rosen JL, et al. Prodromal assessment with the structured interview for prodromal syndromes and the scale of prodromal symptoms: predictive validity, interrater reliability, and training to reliability. *Schizophrenia bulletin* 2003;29(4):703-715.

**23.** Yung AR, Phillips LJ, Yuen HP, Francey SM, McFarlane CA, Hallgren M, McGorry PD. Psychosis prediction: 12-month follow up of a high-risk ("prodromal") group. *Schizophrenia research* Mar 1 2003;60(1):21-32.

**24.** Brewer WJ, Wood SJ, McGorry PD, et al. Impairment of olfactory identification ability in individuals at ultra-high risk for psychosis who later develop schizophrenia. *The American journal of psychiatry* Oct 2003;160(10):1790-1794.

**25.** Lencz T, Smith CW, Auther AM, Correll CU, Cornblatt BA. The assessment of "prodromal schizophrenia": unresolved issues and future directions. *Schizophrenia bulletin* 2003;29(4):717-728.

**26.** Wood SJ, Pantelis C, Proffitt T, et al. Spatial working memory ability is a marker of risk-for-psychosis. *Psychological medicine* Oct 2003;33(7):1239-1247.

**27.** Yung AR, Phillips LJ, Yuen HP, McGorry PD. Risk factors for psychosis in an ultra high-risk group: psychopathology and clinical features. *Schizophrenia research* Apr 1 2004;67(2-3):131-142.

**28.** Mason O, Startup M, Halpin S, Schall U, Conrad A, Carr V. Risk factors for transition to first episode psychosis among individuals with 'at-risk mental states'. *Schizophrenia research* Dec 1 2004;71(2-3):227-237.

**29.** Hafner H, Maurer K, Ruhrmann S, et al. Early detection and secondary prevention of psychosis: facts and visions. *European archives of psychiatry and clinical neuroscience* Apr 2004;254(2):117-128.

**30.** Yung AR, Yuen HP, McGorry PD, et al. Mapping the onset of psychosis: the Comprehensive Assessment of At-Risk Mental States. *The Australian and New Zealand journal of psychiatry* Nov-Dec 2005;39(11-12):964-971.

**31.** Brewer WJ, Francey SM, Wood SJ, et al. Memory impairments identified in people at ultra-high risk for psychosis who later develop first-episode psychosis. *The American journal of psychiatry* Jan 2005;162(1):71-78.

**32.** Francey SM, Jackson HJ, Phillips LJ, Wood SJ, Yung AR, McGorry PD. Sustained attention in young people at high risk of psychosis does not predict transition to psychosis. *Schizophrenia research* Nov 1 2005;79(1):127-136.

**33.** Yun Y, Phillips LJ, Cotton S, Yung AR, Francey SM, Yuen HP, McGorry PD. Obstetric complications and transition to psychosis in an "ultra" high risk sample. *The Australian and New Zealand journal of psychiatry* Jun 2005;39(6):460-466.

**34.** Garner B, Pariante CM, Wood SJ, et al. Pituitary volume predicts future transition to psychosis in individuals at ultra-high risk of developing psychosis. *Biological psychiatry* Sep 1 2005;58(5):417-423.

**35.** Job DE, Whalley HC, McIntosh AM, Owens DG, Johnstone EC, Lawrie SM. Grey matter changes can improve the prediction of schizophrenia in subjects at high risk. *BMC medicine* 2006;4:29.

**36.** Lam MM, Hung SF, Chen EY. Transition to psychosis: 6-month follow-up of a Chinese high-risk group in Hong Kong. *The Australian and New Zealand journal of psychiatry* May 2006;40(5):414-420.

**37.** Lemos S, Vallina O, Fernandez P, et al. [Predictive validity of the Scale of Prodromal Symptoms (SOPS)]. *Actas espanolas de psiquiatria* Jul-Aug 2006;34(4):216-223.

**38.** Keefe RS, Perkins DO, Gu H, Zipursky RB, Christensen BK, Lieberman JA. A longitudinal study of neurocognitive function in individuals at-risk for psychosis. *Schizophrenia research* Dec 2006;88(1-3):26-35.

**39.** Lencz T, Smith CW, McLaughlin D, Auther A, Nakayama E, Hovey L, Cornblatt BA. Generalized and specific neurocognitive deficits in prodromal schizophrenia. *Biological psychiatry* May 1 2006;59(9):863-871.

**40.** Velakoulis D, Wood SJ, Wong MT, et al. Hippocampal and amygdala volumes according to psychosis stage and diagnosis: a magnetic resonance imaging study of chronic schizophrenia, first-episode psychosis, and ultra-high-risk individuals. *Archives of general psychiatry* Feb 2006;63(2):139-149.

**41.** Simon AE, Dvorsky DN, Boesch J, Roth B, Isler E, Schueler P, Petralli C, Umbricht D. Defining subjects at risk for psychosis: a comparison of two approaches. *Schizophrenia research* Jan 1 2006;81(1):83-90.

**42.** Haroun N, Dunn L, Haroun A, Cadenhead KS. Risk and protection in prodromal schizophrenia: ethical implications for clinical practice and future research. *Schizophrenia bulletin* Jan 2006;32(1):166-178.

**43.** Broome MR, Day F, Valli I, Valmaggia L, Johns LC, Howes O, Garety P, McGuire PK. Delusional ideation, manic symptomatology and working memory in a cohort at clinical high-risk for psychosis: a longitudinal study. *European psychiatry : the journal of the Association of European Psychiatrists* May 2012;27(4):258-263.

**44.** Hoffman RE, Woods SW, Hawkins KA, et al. Extracting spurious messages from noise and risk of schizophrenia-spectrum disorders in a prodromal population. *The British journal of psychiatry : the journal of mental science* Oct 2007;191:355-356.

**45.** Kristensen K, Cadenhead KS. Cannabis abuse and risk for psychosis in a prodromal sample. *Psychiatry research* May 30 2007;151(1-2):151-154.

**46.** Thompson KN, Berger G, Phillips LJ, Komesaroff P, Purcell R, McGorry PD. HPA axis functioning associated with transition to psychosis: combined DEX/CRH test. *Journal of psychiatric research* Aug 2007;41(5):446-450.

**47.** Pukrop R, Ruhrmann S, Schultze-Lutter F, Bechdolf A, Brockhaus-Dumke A, Klosterkotter J. Neurocognitive indicators for a conversion to psychosis: comparison of patients in a potentially initial prodromal state who did or did not convert to a psychosis. *Schizophrenia research* May 2007;92(1-3):116-125.

**48.** Broome MR, Johns LC, Valli I, et al. Delusion formation and reasoning biases in those at clinical high risk for psychosis. *The British journal of psychiatry Supplement* Dec 2007;51:s38-42.

**49.** Nieman D, Becker H, van de Fliert R, et al. Antisaccade task performance in patients at ultra high risk for developing psychosis. *Schizophrenia research* Sep 2007;95(1-3):54-60.

**50.** Amminger GP, Wood SJ, Nelson BC, Francey SM, Yung AR, Mackinnon A, Yolken RH, McGorry PD. Association of serum antibodies to herpes simplex virus 1 with cognitive deficits in individuals at ultra-high risk of psychosis with and without subsequent transition. *Early Interv Psychia* Oct 2008;2:A62-A62.

**51.** Borgwardt SJ, McGuire PK, Aston J, Gschwandtner U, Pfluger MO, Stieglitz RD, Radue EW, Riecher-Rossler A. Reductions in frontal, temporal and parietal volume associated with the onset of psychosis. *Schizophrenia research* Dec 2008;106(2-3):108-114.

**52.** Brockhaus-Dumke A, Schultze-Lutter F, Mueller R, Tendolkar I, Bechdolf A, Pukrop R, Klosterkoetter J, Ruhrmann S. Sensory gating in schizophrenia: P50 and N100 gating in antipsychotic-free subjects at risk, first-episode, and chronic patients. *Biological psychiatry* Sep 1 2008;64(5):376-384.

**53.** Cannon TD, Cadenhead K, Cornblatt B, et al. Prediction of psychosis in youth at high clinical risk: a multisite longitudinal study in North America. *Archives of general psychiatry* Jan 2008;65(1):28-37.

**54.** Walder DJ, Mittal V, Trotman HD, McMillan AL, Walker EF. Neurocognition and conversion to psychosis in adolescents at high-risk. *Schizophrenia research* Apr 2008;101(1-3):161-168.

**55.** Walterfang M, Yung A, Wood AG, et al. Corpus callosum shape alterations in individuals prior to the onset of psychosis. *Schizophrenia research* Aug 2008;103(1-3):1-10.

**56.** Hurlemann R, Matusch A, Kuhn KU, et al. 5-HT2A receptor density is decreased in the at-risk mental state. *Psychopharmacology* Jan 2008;195(4):579-590.

**57.** Takahashi T, Yucel M, Yung AR, et al. Adhesio interthalamica in individuals at high-risk for developing psychosis and patients with psychotic disorders. *Progress in neuro-psychopharmacology & biological psychiatry* Oct 1 2008;32(7):1708-1714.

**58.** Keri S, Kiss I, Kelemen O. Effects of a neuregulin 1 variant on conversion to schizophrenia and schizophreniform disorder in people at high risk for psychosis. *Molecular psychiatry* Feb 2009;14(2):118-119.

**59.** Koutsouleris N, Meisenzahl EM, Davatzikos C, et al. Use of neuroanatomical pattern classification to identify subjects in at-risk mental states of psychosis and predict disease transition. *Archives of general psychiatry* Jul 2009;66(7):700-712.

**60.** Koutsouleris N, Schmitt GJ, Gaser C, et al. Neuroanatomical correlates of different vulnerability states for psychosis and their clinical outcomes. *The British journal of psychiatry : the journal of mental science* Sep 2009;195(3):218-226.

**61.** Lemos-Giraldez S, Vallina-Fernandez O, Fernandez-Iglesias P, et al. Symptomatic and functional outcome in youth at ultra-high risk for psychosis: a longitudinal study. *Schizophrenia research* Dec 2009;115(2-3):121-129.

**62.** Mittal VA, Willhite R, Daley M, Bearden CE, Niendam T, Ellman LM, Cannon TD. Obstetric complications and risk for conversion to psychosis among individuals at high clinical risk. *Early Interv Psychia* Aug 2009;3(3):226-230.

**63.** Riecher-Rössler A, Pflueger MO, Aston J, Borgwardt SJ, Brewer WJ, Gschwandtner U, Stieglitz RD. Efficacy of using cognitive status in predicting psychosis: a 7-year follow-up. *Biological psychiatry* Dec 1 2009;66(11):1023-1030.

**64.** Salokangas RK, Heinimaa M, Svirskis T, et al. Perceived negative attitude of others as an early sign of psychosis. *European psychiatry : the journal of the Association of European Psychiatrists* May 2009;24(4):233-238.

**65.** Sun D, Phillips L, Velakoulis D, et al. Progressive brain structural changes mapped as psychosis develops in 'at risk' individuals. *Schizophrenia research* Mar 2009;108(1-3):85-92.

**66.** Velthorst E, Nieman DH, Becker HE, et al. Baseline differences in clinical symptomatology between ultra high risk subjects with and without a transition to psychosis. *Schizophrenia research* Apr 2009;109(1-3):60-65.

**67.** Miyakoshi T, Matsumoto K, Ito F, Ohmuro N, Matsuoka H. Application of the Comprehensive Assessment of At-Risk Mental States (CAARMS) to the Japanese population: reliability and validity of the Japanese version of the CAARMS. *Early Interv Psychia* May 2009;3(2):123-130.

**68.** Takahashi T, Wood SJ, Yung AR, et al. Insular cortex gray matter changes in individuals at ultra-high-risk of developing psychosis. *Schizophrenia research* Jun 2009;111(1-3):94-102.

**69.** Fusar-Poli P, Meneghelli A, Valmaggia L, Allen P, Galvan F, McGuire P, Cocchi A. Duration of untreated prodromal symptoms and 12-month functional outcome of individuals at risk of psychosis. *The British journal of psychiatry : the journal of mental science* Feb 2009;194(2):181-182.

**70.** Simon AE, Umbricht D. High remission rates from an initial ultra-high risk state for psychosis. *Schizophrenia research* Feb 2010;116(2-3):168-172.

**71.** Bloemen OJ, de Koning MB, Schmitz N, et al. White-matter markers for psychosis in a prospective ultra-high-risk cohort. *Psychological medicine* Aug 2010;40(8):1297-1304.

**72.** Bechdolf A, Thompson A, Nelson B, et al. Experience of trauma and conversion to psychosis in an ultra-high-risk (prodromal) group. *Acta psychiatrica Scandinavica* May 2010;121(5):377-384.

**73.** Fusar-Poli P, Byrne M, Valmaggia L, Day F, Tabraham P, Johns L, McGuire P, Team O. Social dysfunction predicts two years clinical outcome in people at ultra high risk for psychosis. *Journal of psychiatric research* Apr 2010;44(5):294-301.

**74.** Korver N, Nieman DH, Becker HE, et al. Symptomatology and neuropsychological functioning in cannabis using subjects at ultra-high risk for developing psychosis and healthy controls. *The Australian and New Zealand journal of psychiatry* Mar 2010;44(3):230-236.

**75.** Mittal VA, Walker EF, Bearden CE, Walder D, Trottman H, Daley M, Simone A, Cannon TD. Markers of basal ganglia dysfunction and conversion to psychosis: neurocognitive deficits and dyskinesias in the prodromal period. *Biological psychiatry* Jul 1 2010;68(1):93-99.

**76.** Mossner R, Schuhmacher A, Wagner M, et al. DAOA/G72 predicts the progression of prodromal syndromes to first episode psychosis. *European archives of psychiatry and clinical neuroscience* Apr 2010;260(3):209-215.

**77.** Nelson B, Yung AR. Can clinicians predict psychosis in an ultra high risk group? *The Australian and New Zealand journal of psychiatry* Jul 2010;44(7):625-630.

**78.** Olvet DM, Stearns WH, McLaughlin D, Auther AM, Correll CU, Cornblatt BA. Comparing clinical and neurocognitive features of the schizophrenia prodrome to the bipolar prodrome. *Schizophrenia research* Oct 2010;123(1):59-63.

**79.** Ruhrmann S, Schultze-Lutter F, Salokangas RK, et al. Prediction of psychosis in adolescents and young adults at high risk: results from the prospective European prediction of psychosis study. *Archives of general psychiatry* Mar 2010;67(3):241-251.

**80.** Schlosser DA, Zinberg JL, Loewy RL, Casey-Cannon S, O'Brien MP, Bearden CE, Vinogradov S, Cannon TD. Predicting the longitudinal effects of the family environment on prodromal symptoms and functioning in patients at-risk for psychosis. *Schizophrenia research* May 2010;118(1-3):69-75.

**81.** Jung MH, Jang JH, Kang DH, et al. The reliability and validity of the korean version of the structured interview for prodromal syndrome. *Psychiatry investigation* Dec 2010;7(4):257-263.

**82.** Sabb FW, van Erp TG, Hardt ME, Dapretto M, Caplan R, Cannon TD, Bearden CE. Language network dysfunction as a predictor of outcome in youth at clinical high risk for psychosis. *Schizophrenia research* Feb 2010;116(2-3):173-183.

**83.** Seidman LJ, Giuliano AJ, Meyer EC, et al. Neuropsychology of the prodrome to psychosis in the NAPLS consortium: relationship to family history and conversion to psychosis. *Archives of general psychiatry* Jun 2010;67(6):578-588.

**84.** Ziermans TB, Schothorst PF, Sprong M, van Engeland H. Transition and remission in adolescents at ultra-high risk for psychosis. *Schizophrenia research* Mar 2011;126(1-3):58-64.

**85.** Bearden CE, Wu KN, Caplan R, Cannon TD. Thought disorder and communication deviance as predictors of outcome in youth at clinical high risk for psychosis. *Journal of the American Academy of Child and Adolescent Psychiatry* Jul 2011;50(7):669-680.

**86.** Dragt S, Nieman DH, Veltman D, Becker HE, van de Fliert R, de Haan L, Linszen DH. Environmental factors and social adjustment as predictors of a first psychosis in subjects at ultra high risk. *Schizophrenia research* Jan 2011;125(1):69-76.

**87.** Fontenelle LF, Lin A, Pantelis C, Wood SJ, Nelson B, Yung AR. A longitudinal study of obsessive-compulsive disorder in individuals at ultra-high risk for psychosis. *Journal of psychiatric research* Sep 2011;45(9):1140-1145.

**88.** Raballo A, Nelson B, Thompson A, Yung A. The comprehensive assessment of at-risk mental states: from mapping the onset to mapping the structure. *Schizophrenia research* Apr 2011;127(1-3):107-114.

**89.** Simeonova DI, Attalla A, Trotman H, Esterberg M, Walker EF. Does a parent-report measure of behavioral problems enhance prediction of conversion to psychosis in clinical high-risk adolescents? *Schizophrenia research* Aug 2011;130(1-3):157-163.

**90.** Thompson A, Nelson B, Yung A. Predictive validity of clinical variables in the "at risk" for psychosis population: international comparison with results from the North American Prodrome Longitudinal Study. *Schizophrenia research* Mar 2011;126(1-3):51-57.

**91.** Velthorst E, Nieman DH, Klaassen RM, Becker HE, Dingemans PM, Linszen DH, De Haan L. Three-year course of clinical symptomatology in young people at ultra high risk for transition to psychosis. *Acta psychiatrica Scandinavica* Jan 2011;123(1):36-42.

**92.** Kim HS, Shin NY, Jang JH, Kim E, Shim G, Park HY, Hong KS, Kwon JS. Social cognition and neurocognition as predictors of conversion to psychosis in individuals at ultra-high risk. *Schizophrenia research* Aug 2011;130(1-3):170-175.

**93.** Schimmelmann BG, Michel C, Schaffner N, Schultze-Lutter F. What percentage of people in the general population satisfies the current clinical at-risk criteria of psychosis? *Schizophrenia research* Jan 2011;125(1):99-100.

**94.** Addington J, Cornblatt BA, Cadenhead KS, et al. At clinical high risk for psychosis: outcome for nonconverters. *The American journal of psychiatry* Aug 2011;168(8):800-805.

**95.** Demjaha A, Valmaggia L, Stahl D, Byrne M, McGuire P. Disorganization/cognitive and negative symptom dimensions in the at-risk mental state predict subsequent transition to psychosis. *Schizophrenia bulletin* Mar 2012;38(2):351-359.

**96.** Dragt S, Nieman DH, Schultze-Lutter F, et al. Cannabis use and age at onset of symptoms in subjects at clinical high risk for psychosis. *Acta psychiatrica Scandinavica* Jan 2012;125(1):45-53.

**97.** Fusar-Poli P, Hobson R, Raduelli M, Balottin U. Reliability and validity of the Comprehensive Assessment of the At Risk Mental State, Italian version (CAARMS-I). *Current pharmaceutical design* 2012;18(4):386-391.

**98.** Koutsouleris N, Davatzikos C, Bottlender R, et al. Early recognition and disease prediction in the at-risk mental states for psychosis using neurocognitive pattern classification. *Schizophrenia bulletin* Nov 2012;38(6):1200-1215.

**99.** Schlosser DA, Jacobson S, Chen Q, Sugar CA, Niendam TA, Li G, Bearden CE, Cannon TD. Recovery from an at-risk state: clinical and functional outcomes of putatively prodromal youth who do not develop psychosis. *Schizophrenia bulletin* Nov 2012;38(6):1225-1233.

**100.** Bechdolf A, Wagner M, Ruhrmann S, et al. Preventing progression to first-episode psychosis in early initial prodromal states. *The British journal of psychiatry : the journal of mental science* Jan 2012;200(1):22-29.

**101.** Carrion RE, McLaughlin D, Goldberg TE, Auther AM, Olsen RH, Olvet DM, Correll CU, Cornblatt BA. Prediction of functional outcome in individuals at clinical high risk for psychosis. *JAMA psychiatry* Nov 2013;70(11):1133-1142.

**102.** Fusar-Poli P, Byrne M, Badger S, Valmaggia LR, McGuire PK. Outreach and support in south London (OASIS), 2001-2011: ten years of early diagnosis and treatment for young individuals at high clinical risk for psychosis. *European psychiatry : the journal of the Association of European Psychiatrists* Jun 2013;28(5):315-326.

**103.** Nelson B, Yuen HP, Wood SJ, et al. Long-term follow-up of a group at ultra high risk ("prodromal") for psychosis: the PACE 400 study. *JAMA psychiatry* Aug 2013;70(8):793-802.

**104.** Nieman DH, Ruhrmann S, Dragt S, et al. Psychosis Prediction: Stratification of Risk Estimation With Information-Processing and Premorbid Functioning Variables. *Schizophrenia bulletin* Oct 18 2013.

**105.** Nieman DH, Velthorst E, Becker HE, et al. The Strauss and Carpenter Prognostic Scale in subjects clinically at high risk of psychosis. *Acta psychiatrica Scandinavica* Jan 2013;127(1):53-61.

**106.** Tarbox SI, Addington J, Cadenhead KS, et al. Premorbid functional development and conversion to psychosis in clinical high-risk youths. *Development and psychopathology* Nov 2013;25(4 Pt 1):1171-1186.

**107.** Chung YC, Kang NI, Im YJ, Kim SW, Cho IH, Lee YM, Kwon JS. Validation of the Korean version of the Eppendorf Schizophrenia Inventory as a screening measure to detect adolescents at ultra-high risk for psychosis. *Early Interv Psychia* Feb 2013;7(1):71-79.

**108.** Buchy L, Perkins D, Woods SW, Liu L, Addington J. Impact of substance use on conversion to psychosis in youth at clinical high risk of psychosis. *Schizophrenia research* Jul 2014;156(2-3):277-280.

**109.** DeVylder JE, Muchomba FM, Gill KE, Ben-David S, Walder DJ, Malaspina D, Corcoran CM. Symptom trajectories and psychosis onset in a clinical high-risk cohort: The relevance of subthreshold thought disorder. *Schizophrenia research* Sep 19 2014.

**110.** Manninen M, Lindgren M, Therman S, Huttunen M, Ebeling H, Moilanen I, Suvisaari J. Clinical high-risk state does not predict later psychosis in a delinquent adolescent population. *Early Interv Psychia* Feb 2014;8(1):87-90.

**111.** Tay SA, Yuen S, Lim LK, Pariyasami S, Rao S, Poon LY, Verma S. Support for Wellness Achievement Programme (SWAP): clinical and demographic characteristics of young people with at-risk mental state in Singapore. *Early Interv Psychia* Sep 9 2014.

**112.** Katsura M, Ohmuro N, Obara C, Kikuchi T, Ito F, Miyakoshi T, Matsuoka H, Matsumoto K. A naturalistic longitudinal study of at-risk mental state with a 2.4 year follow-up at a specialized clinic setting in Japan. *Schizophrenia research* Sep 2014;158(1-3):32-38.

**113.** Koutsouleris N, Davatzikos C, Borgwardt S, et al. Accelerated brain aging in schizophrenia and beyond: a neuroanatomical marker of psychiatric disorders. *Schizophrenia bulletin* Sep 2014;40(5):1140-1153.

**114.** Lindgren M, Manninen M, Kalska H, et al. Predicting psychosis in a general adolescent psychiatric sample. *Schizophrenia research* Sep 2014;158(1-3):1-6.

**115.** Modinos G, Allen P, Frascarelli M, et al. Are we really mapping psychosis risk? Neuroanatomical signature of affective disorders in subjects at ultra high risk. *Psychological medicine* Dec 2014;44(16):3491-3501.

**116.** Theodoridou A, Heekeren K, Dvorsky D, et al. Early Recognition of High Risk of Bipolar Disorder and Psychosis: An Overview of the ZInEP "Early Recognition" Study. *Frontiers in public health* 2014;2:166.

**117.** Thompson AD, Nelson B, Yuen HP, Lin A, Amminger GP, McGorry PD, Wood SJ, Yung AR. Sexual trauma increases the risk of developing psychosis in an ultra high-risk "prodromal" population. *Schizophrenia bulletin* May 2014;40(3):697-706.

**118.** van Tricht MJ, Ruhrmann S, Arns M, et al. Can quantitative EEG measures predict clinical outcome in subjects at Clinical High Risk for psychosis? A prospective multicenter study. *Schizophrenia research* Mar 2014;153(1-3):42-47.

**119.** Chen F, Wang L, Wang J, Heeramun-Aubeeluck A, Yuan J, Zhao X. Applicability of the Chinese version of the 16-item Prodromal Questionnaire (CPQ-16) for identifying attenuated psychosis syndrome in a college population. *Early intervention in psychiatry* Aug 12 2014.

**120.** Braham A, Bannour AS, Ben Romdhane A, Nelson B, Bougumiza I, Ben Nasr S, Elkissi Y, Ben Hadj Ali B. Validation of the Arabic version of the Comprehensive Assessment of At Risk Mental States (CAARMS) in Tunisian adolescents and young adults. *Early Interv Psychia* May 2014;8(2):147-154.

**121.** Michel C, Ruhrmann S, Schimmelmann BG, Klosterkotter J, Schultze-Lutter F. A Stratified Model for Psychosis Prediction in Clinical Practice. *Schizophrenia bulletin* Mar 7 2014.

**122.** Woods SW, Walsh BC, Addington J, et al. Current status specifiers for patients at clinical high risk for psychosis. *Schizophrenia research* Sep 2014;158(1-3):69-75.

**123.** Ramyead A, Kometer M, Studerus E, Koranyi S, Ittig S, Gschwandtner U, Fuhr P, Riecher-Rossler A. Aberrant Current Source-Density and Lagged Phase Synchronization of Neural Oscillations as Markers for Emerging Psychosis. *Schizophrenia bulletin* Sep 9 2014.

**124.** Perkins DO, Jeffries CD, Addington J, et al. Towards a Psychosis Risk Blood Diagnostic for Persons Experiencing High-Risk Symptoms: Preliminary Results From the NAPLS Project. *Schizophrenia bulletin* Aug 6 2014.

**125.** Ziermans T, de Wit S, Schothorst P, Sprong M, van Engeland H, Kahn R, Durston S. Neurocognitive and clinical predictors of long-term outcome in adolescents at ultra-high risk for psychosis: a 6-year follow-up. *PloS one* 2014;9(4):e93994.

**126.** Poe SL, Gill KE, Brucato G, Corcoran CM, Girgis RR. Family history of psychosis as a predictor or protective factor of social maladjustment in a population at clinical high risk for psychosis. *Psychiatry research* Nov 30 2014;219(3):696-699.
